# Feasibility Assessment of Extracting Social Determinants of Health From Electronic Medical Records: A Multicenter Study Across Chinese Healthcare Institutions

**DOI:** 10.1002/hcs2.70094

**Published:** 2026-08-31

**Authors:** Mengchun Gong, Zihao Ouyang, Dandan Ma, Qilin Wang, Endi Cai, Chao Liu, Yue Yu, Jingdong Yan, Sheng Nie, Lin Lin

**Affiliations:** ^1^ School of Biomedical Engineering, Guangdong Medical University Dongguan China; ^2^ Vice President's Office DHC Technologies Ltd. Beijing China; ^3^ National Clinical Research Center for Aging Fudan University Affiliated Huashan Hospital Shanghai China; ^4^ Medical Research Department DHC Technologies Ltd. Beijing China; ^5^ Department of Information Management, State Key Laboratory of Complex Severe and Rare Diseases Peking Union Medical College Hospital, Chinese Academy of Medical Sciences and Peking Union Medical College Beijing China; ^6^ Department of Nephrology, Nanfang Hospital Southern Medical University Guangzhou China; ^7^ Department of Biomedical Engineering, School of Biomedical Engineering Guangdong Medical University Dongguan China; ^8^ Key Laboratory of Medical Electronics and Medical Imaging Equipment Guangdong Medical University Dongguan China; ^9^ Guangdong Medical University Songshan Lake Innovation Center of Medicine & Engineering Guangdong Medical University Dongguan China

**Keywords:** Chinese electronic medical record, natural language processing, real‐world data, social determinants of health

## Abstract

**Background:**

Social determinants of health (SDoH) are pivotal in influencing health outcomes and disparities across various populations. Real‐world data rich in SDoH information, such as electronic medical records (EMRs), can considerably enhance public health interventions. However, in Chinese medical practice, these non‐clinical factors are often neglected, with many healthcare providers failing to recognize the importance of SDoH information in the improvement of patient care. The objective of this study is to explore the feasibility and effectiveness of extracting SDoH information from Chinese EMRs.

**Methods:**

We developed a China‐specific SDoH classification framework by integrating findings from relevant research using real‐world data. This framework was applied to over three million patient records from Chinese EMRs to examine the completeness and availability of SDoH‐related attributes within relevant fields. We also developed a quantitative assessment framework for evaluating SDoH information in EMR fields. This two‐dimensional evaluation system measures data completeness and availability using a three‐level hierarchical scoring approach, progressing from basic to advanced criteria. Additionally, we analyzed variations in SDoH information extraction across different healthcare institutions.

**Results:**

Drawing on the literature and 2000 manually annotated EMRs, we established a standardized framework of SDoH factors, comprising 50 features tailored to the Chinese medical diagnostic and treatment environment. We analyzed over 5.6 million EMRs from 40 hospitals within the National Clinical Research Data Center and found that tables and fields in Chinese EMRs cover all six primary SDoH categories, encompassing 25 out of 50 specific attributes. However, data extraction feasibility was relatively poor, with only seven features being fully extractable, with most “social and community context” data missing. Our evaluation of 43 electronic health record fields containing SDoH data revealed significant disparities between completeness and availability metrics. The composite completeness score averaged 1.47 (95% confidence interval: 1.20–1.73) out of a maximum score of 3. Availability assessments demonstrated notably higher performance with a mean score of 2.14 (95% confidence interval: 1.83–2.44) out of a maximum score of 3.

**Conclusions:**

This research established a culturally adapted SDoH framework for China and demonstrated the feasibility of extracting SDoH attributes from Chinese EMRs. Although some SDoH information in EMRs still cannot be captured or requires more advanced data processing to be usable, Chinese EMRs contain a wealth of SDoH data, allowing us to use large existing EMR databases to support ongoing SDoH research. Natural language processing technology has a critical role in this process, underscoring the importance of medical informatics and current artificial intelligence techniques in medicine and public health. Our research lays a foundation for future SDoH studies in China, enabling more comprehensive research and encouraging the government and relevant agencies to focus on SDoH interventions.

## Graphical abstract

We developed a China‐specific social determinants of health (SDoH) framework and extracted SDoH information from a database of 5.6 million electronic medical records (EMRs) across more than 40 centers. The results showed that these EMRs include extractable data covering some SDoH categories, with moderate completeness and high availability.

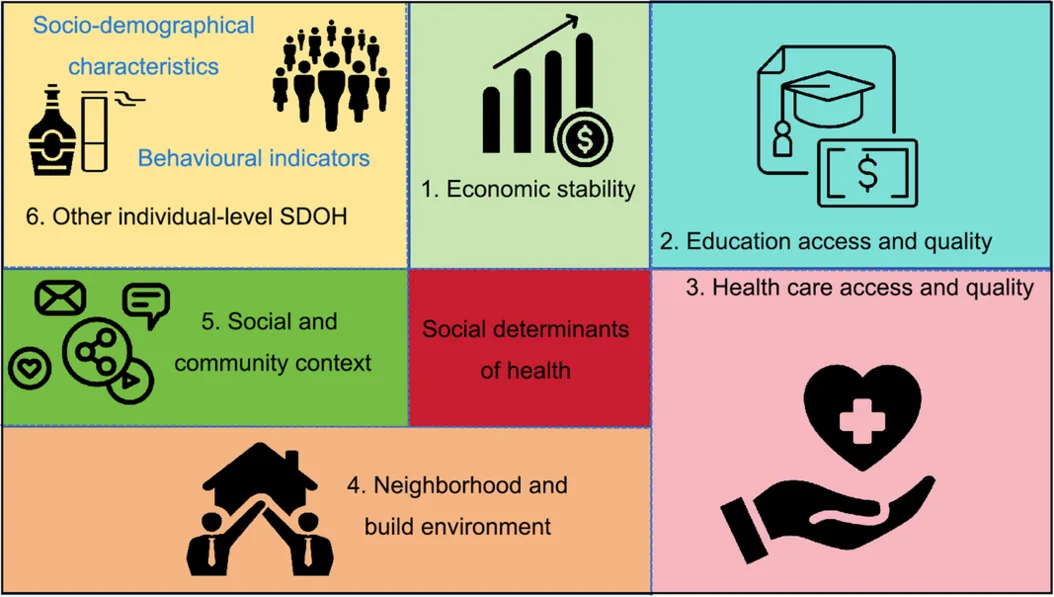

**Abbreviations**
BERT: Bidirectional Encoder Representations from Transformers
CRDS: Chinese Renal Disease System
EHR: electronic health record
EMR: electronic medical record
LLM: large language model
NLP: natural language processing
RWD: real‐world data
SDoH: social determinants of health
WHO: World Health Organization

## 1 Background

Social determinants of health (SDoH) are defined by the World Health Organization (WHO) as “nonmedical factors that influence health outcomes,” encompassing “the conditions in which people are born, grow, live, work, and age” [1]. SDoH arise from various social, political, and economic forces, contributing to individual factors that greatly affect health status, such as income, education, and culture [2]. In the past decade, a rapidly growing body of literature has demonstrated the important role of SDoH in shaping human health and well‐being [3]. In the United States, SDoH are estimated to contribute to up to 40% of all preventable deaths, a figure that far exceeds the 10%–15% attributed to improvements in medical care [4]. Similarly, in Europe, the impact of SDoH on disease management is gaining recognition, with adverse elements like low socioeconomic status and poor living conditions significantly increasing the risk of cardiovascular and cerebrovascular diseases [5]. During patient care, healthcare providers consider SDoH in screening and treatment, which in turn affects treatment outcomes [6]. Consequently, the systematic collection, organization, and analysis of SDoH data are essential for accurate diagnosis, effective treatment, and comprehensive healthcare management [6].

In recent years, owing to the rapid development of artificial intelligence, it has become increasingly possible to gain deeper insights using text records within patients' electronic medical records (EMRs) and using natural language processing (NLP) techniques [7]. At the same time, an increasing number of studies have used real‐world data (RWD) to explore SDoH in public and population health research, with a focus on sources like EMRs [8, 9], public health survey data (e.g. the National Health and Nutrition Examination Survey), and other data such as exposome data [10]. The integration of SDoH into disease screening and prediction models facilitates precision prevention and treatment by identifying social risks. Importantly, SDoH‐enriched RWD can also support public health interventions by identifying populations at higher risk for specific health problems, enabling more targeted and effective interventions for early prevention [11]. Understanding the influence of social and environmental factors on health and healthcare access allows health systems and providers to collaborate with communities and public health organizations to address these factors, reduce disparities, and enhance health equity. Because SDoH are often the root causes of disparities and account for a considerable proportion of modifiable factors, it is challenging to identify and link SDoH information with clinical and public health data [12, 13]. Nevertheless, informatics approaches such as NLP, ontologies, and spatiotemporal data integration offer promising solutions to these challenges [14].

In China, there are relatively few studies on SDoH based on RWD, particularly those concerning the mining of SDoH‐related factors from EMRs. One factor contributing to this situation is that the structure of Chinese EMRs is primarily designed for clinical use, making it challenging to effectively extract SDoH information. This leads to inadequate attention paid to SDoH in Chinese hospitals, which results in failure to consider this crucial factor during the process of patient care. Xu et al. note that despite guidelines, Chinese medical records contain inconsistencies and errors owing to poor recordkeeping habits or knowledge [15]. Moreover, extracting structured data from Chinese EMRs is difficult owing to denser information in the Chinese language as compared with English [16]. However, the impact of SDoH on the life expectancy of the Chinese population should not be underestimated [17].

In this study, we aimed to identify Chinese SDoH factors alongside the RWD of hospital EMRs and to explore the feasibility and effectiveness of extracting SDoH attributes from Chinese EMRs. We developed a set of SDoH information analysis models suitable for the Chinese context. Using these models, we aimed to assist healthcare providers in making more accurate diagnoses, thereby improving the overall level of healthcare delivery.

## 2 Methods

### 2.1 Aims and Objectives

Our goal was to establish a China‐specific SDoH factor catalog and to extract SDoH factors from a large number of Chinese EMRs using information technology. This approach maximizes the use of existing data and enables the assessment of China's healthcare landscape based on extracted SDoH outcomes, to ultimately enhance overall medical service quality.

### 2.2 Data Source

The National Clinical Research Data Center has established a cooperative alliance with over 40 renowned hospitals nationwide, including the Affiliated Hospital of Southern Medical University and the Affiliated Children's Hospital of Fudan University, among others. Through this collaboration, a considerable amount of Chinese EMR data has been amassed, including detailed descriptions of patients' diseases, medication histories, and comprehensive outcomes of various medical tests and procedures. Among all records, data concerning kidney‐related diseases are the most comprehensive, involving 3,324,181 patients and 5,651,133 treatment records.

### 2.3 The Chinese Renal Disease System (CRDS) Database

The CRDS database aggregates and integrates EMR data from over 40 hospitals, establishing a data structure that is suitable for clinical research. The platform is tailored based on data quality and research needs, resulting in a patient‐centered data system. The structure is divided into patient visit information and clinical diagnosis and treatment information, with all case text data stored uniformly in the Note table. We selectively structured these text data to facilitate use by researchers. For critical text data elements, the database has implemented NLP‐enabled structured preprocessing, systematically organizing clinical text data into nine standardized NLP tables: chiefComplaint, drinkSmokeHistory, toxicHistory, diseaseName, diseaseOperation, allergy, familyHistory, marriageHistory, and vital_Signs. This data system offers two main advantages: it standardizes the complex structure of Chinese EMRs, making data searching and use more efficient, and it structures key text data into readily usable fields. To support the extraction of SDoH‐related information from Chinese EMRs, this system significantly enhances research efficiency (Table S1).

### 2.4 Chinese SDoH Framework

Current SDoH frameworks are largely based on the WHO's five‐category model. However, this international definition does not fully align with China's healthcare context. Through systematic reviews of China‐specific health surveys and the academic literature, and rigorous examination of 2000 comprehensive data samples extracted from the CRDS database, we developed an SDoH classification tailored to the Chinese context. We also consulted relevant experts for recommendations and validation of our classification results.

### 2.5 NLP Implementation for EMRs

We performed dedicated structuring of the text within admission records. For this, we used a Bidirectional Encoder Representations from Transformers (BERT) model for the structuring task, which is divided into two parts: entity extraction and relationship recognition.

Training samples for the entity model were constructed using a span‐based approach, where each span within a set maximum range is mapped to an entity and its corresponding label. After vectorizing the text with BERT, we obtained vectors indicating the start and end positions of entities, as well as the span vector. These three vectors were concatenated and passed through a linear layer. To predict the score for each entity, we applied a softmax function followed by normalization to the output of the linear layer, ensuring that the sum of the scores for each entity label equals 1. The entity with the highest score is the predicted entity. We used a scoring mechanism to label a small subset of samples, trained a model, and used it to predict a large volume of text, extracting entities and relationships with low prediction scores. A portion of this text was then manually reviewed. We combined these data with a random selection of the previously trained model's data to fine‐tune the model. Through several iterations, the proportion of low‐score data decreases, reducing the need for annotators to label all low‐score data and thereby enhancing their efficiency. Finally, we could effectively collect this type of information.

The core principle of a relational model is to classify each entity pair. This model focuses on adding a boundary and entity type as identifiers before and after the entity span, which is then sent to the BERT model as input to the relationship model. The final classification of the entity relationship occurs in the linear layer. Because this method classifies each entity pair, it encodes the same text multiple times, resulting in substantial computational overhead. To mitigate this, negative sampling is performed during data construction to increase the model's training speed. Additionally, the number of negative samples can be set as a hyperparameter, allowing us to reduce the proportion of negative samples and further improve the training speed. This approach can potentially increase the training speed by three to six times during the actual production process. Similarly, the entity model's scoring mechanism can be used, where each entity pair is scored during the prediction process. The final score indicates the accuracy of the model's prediction.

### 2.6 SDoH Information in EMRs

Using the SDoH framework outlined above as a reference, we searched for relevant fields in the data platform tables, identifying those likely to contain SDoH information. Special focus was given to fields previously processed using NLP to examine the presence of SDoH attributes. Experienced clinical and medical informatics experts then mapped potential SDoH‐related EMR fields based on field descriptions and expertise, creating a corresponding SDoH‐EMR table.

### 2.7 Feasibility of Extracting SDoH Information From EMRs

Following the SDoH‐EMR mapping table, we extracted representative raw data samples from electronic health records (EHRs) of over three million patients across more than 40 participating centers. A fully randomized selection process was applied to EHR tables specified in the mapping protocol, with 200 entries randomly drawn from each table. To ensure methodological rigor, two domain experts conducted blinded dual independent reviews through manual annotation, establishing collective consensus to evaluate the quality of captured SDoH variables. Quality was evaluated along two dimensions: information completeness and information availability, each categorized into three levels—partially complete/partially available, mostly complete/mostly available, and fully complete/fully available. When expert reviews yielded consensus, the results were retained. In instances of disagreement, the lower‐level assessment was selected as the final determination. This approach enabled quantitative assessment of the feasibility and efficiency of extracting SDoH from Chinese EMRs.

### 2.8 Extraction and Analysis of SDoH Information

After finding that relevant SDoH information could be extracted from EMRs, we attempted to extract data using the entire database from fields with high completeness and availability of the extracted information. The analysis was carried out in terms of data comparison among different centers.

### 2.9 Ethical Considerations

This retrospective multicentre study used de‐identified EMR data. The study protocol was reviewed and approved by the Medical Ethics Committee of Nanfang Hospital, Southern Medical University (approval no. NFEC‐2019‐213), which waived the requirement for individual informed consent. All personal identifiers were removed before analysis, and the data were securely stored and accessed only by authorized personnel. No attempts were made to re‐identify individuals, and all results are reported in aggregate to protect patient confidentiality.

## 3 Results

### 3.1 Creating the China SDoH Factor Table

**Figure 1:**
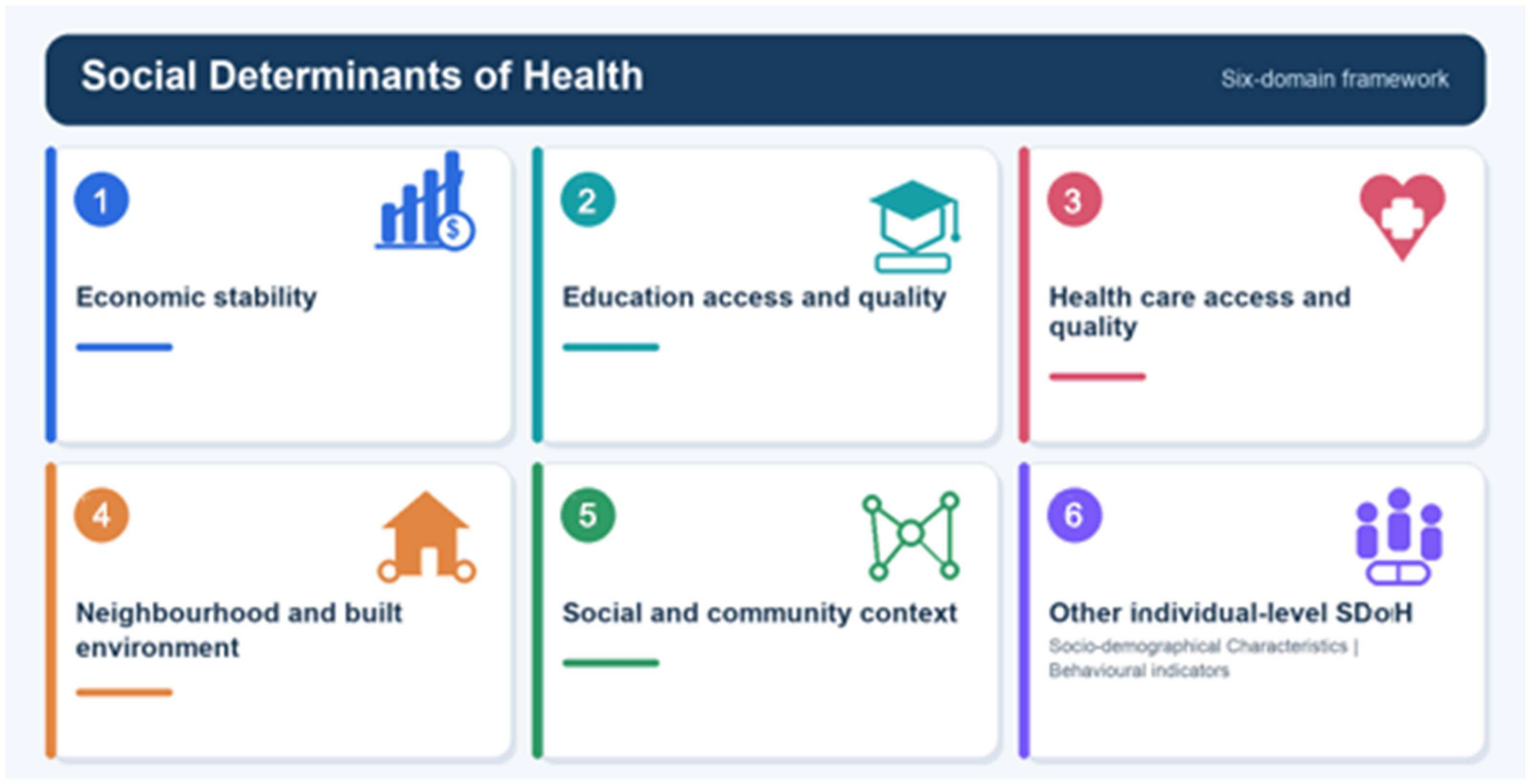
Overview of six different SDoH factors. SDoH, social determinants of health.

The WHO categorizes SDoH into five crucial domains: Economic stability, Accessibility and quality of education, Accessibility and quality of health services, Neighborhood and built environment, and Social and community context [18]. Nevertheless, this international definition is not fully compatible with Chinese medical practice. Therefore, the establishment of an SDoH conceptual framework tailored to China based on the actual circumstances in the country is worth further exploration. Many studies have affirmed that obesity, smoking, alcohol consumption, a high‐salt and high‐fat diet, and other adverse lifestyle choices are major risk factors for chronic diseases [19, 20, 21, 22]. Chronic diseases are thus generally regarded as diseases resulting from an unhealthy lifestyle, the consequence of personal lifestyle decisions. In the SDoH factors table we constructed, personal factors hold an extremely important position [23]. Based on the aforementioned principles and in combination with the professional suggestions of several medical practitioners, we proposed a research framework for SDoH that is suitable for China's national context (Figure 1).

To address the issue of inadequate attention devoted to extracting and analyzing SDoH in Chinese texts, according to this framework, we initially compiled a set of SDoH factors applicable to the Chinese medical clinical context by referring to the published literature and conducting expert consultations [19, 20, 24, 25, 26]. Currently, standardization and normalization in the collection of SDoH information in China's medical and health fields are deficient. In response to this issue, we propose that SDoH information within the healthcare delivery system should be fully exploited to alleviate data scarcity, incomplete information, and difficult access. Given the absence of a uniform measurement of socioeconomic status or standing, we analyzed the association between a series of social structural factors that reflect an individual's economic capacity and health outcomes. On this basis, and combined with our manually annotated data from 2000 medical texts, we ultimately developed a standard framework of SDoH factors adapted to the Chinese medical diagnosis and treatment environment, encompassing 6 types, 21 subtypes, and 50 features (Table S2).

We expanded the WHO‐recommended five domains into six, primarily by incorporating individual‐level SDoH information, including demographic characteristics such as sex and ethnicity, and also encompassing personal health behaviors like smoking and alcohol consumption. These elements demonstrate strong correlations with health outcomes and are readily extractable from EHRs. For the original five domains, we implemented contextual adaptations to better align with China's sociocultural landscape. A notable example is our tripartite categorization of educational attainment into early‐stage education, high school completion, and 4‐year university degree, a structure designed to enhance clinical operability during implementation.

### 3.2 SDoH Information in Chinese EMRs

For the CRDS database, two medical and informatics experts assessed whether the table structures and fields contained the SDoH attributes listed above. This is reflected in the following SDoH‐EMRs map table.

We categorized the EMR structure into 10 major categories: personal information, visit information, diagnosis information, medication orders, test information, surgical information, imaging examination information, death information, medical record text, and physical signs information. Each category was then evaluated to determine if it included the 21 subtypes of SDoH. Some results are presented in Table 1 below. The complete data are available in Supporting File S1: EMRs_SDoH.

**Table 1:** SDoH‐EMRs map table.

		EMRs tables^a^	
SDoH type	Subtype	Person	Visit
1. Economic stability	Income	1	1
	Job benefits	1	0
	Nutrition and healthy eating	1	0
	Housing instability	1	0
	Poverty	1	1
2. Education access and quality	Early childhood development and education	1	0
	High school graduation	1	0
	Enrollment in higher education	1	0
	Language and literacy	0	0
3. Healthcare access and quality	Access to primary care	1	0
	Access to health services	1	0
4. Neighborhood and built environment	Access to foods that support health dietary pattern	1	0
	Environmental conditions	1	0
	Quality of housing	1	0
	Crime and violence	0	0
5. Social and community context	Discrimination	0	0
	Civic participation	0	0
	Social cohesion	0	0
	Incarceration	0	0
6. Other individual‐level SDoH	Sociodemographical characteristics	1	0
	Behavioral indicators	0	0

Abbreviations: EMR, electronic medical record; SDoH, social determinants of health.

^a^ The number “1” means that the table may contain the corresponding information with the subtype.

From the complete table, it can be seen that only a few tables may contain the required SDoH information, mainly concentrated in the Person, Visit, and Note tables. Although the Note table (which contains a large amount of text information) may encompass all required SDoH types, the other relevant tables mainly reflect five major SDoH categories: Economic stability, Education access and quality, Healthcare access and quality, Neighborhood and built environment, and Social and community context. Information related to Social and community context is generally absent.

For the nine NLP tables, only five subtypes (belonging to four categories) can be covered, specifically: Nutrition and healthy eating, Crime and violence, Social cohesion, Sociodemographical characteristics, and Behavioral indicators (Supporting File S1: EMRs(NLP)_SDoH).

Overall, except for the Note table, the EMRs may contain 17 of the 21 SDoH subtypes. The remaining four subtypes—Language and literacy, Discrimination, Civic participation, and Incarceration—are not included. This demonstrates the feasibility of extracting the necessary SDoH information from EMRs.

### 3.3 Feasibility of Extracting SDoH Information From EMRs

For the Person, Visit, and Signs tables, as well as the NLP tables (drinkSmokeHistory, toxicHistory, allergy, familyHistory, marriageHistory, and vital_Signs), we randomly selected 200 records from each table to assess the SDoH information. The assessment was conducted across the following two dimensions.

1. Information completeness: This dimension evaluates whether the fields or data in the table can fully describe a specific feature from the 50 listed SDoH features. We classified the completeness into three levels: partially, mostly, and fully complete.
2. Information availability: This dimension assesses whether the data in the table can be directly used to represent the SDoH feature. The availability was rated according to three levels: requiring multiple conversions and complex technical processes, requiring one or two conversions or simple technical processes, and directly usable without further processing.

The complete data are available in Supporting File S1: Person to vital_Signs.

Through this information extraction process, we found that the tables and fields in Chinese EMRs cover all six major SDoH categories and 25 of the 50 features, accounting for 50% of features. Most of the features that are not covered are concentrated in the Social and community context category. Overall, the average completeness value of the SDoH features in EMR fields is 1.47 (partial completeness), and the average availability is 2.14 (moderately available).

In detail, the following seven features—without health insurance, without access to early education programs, without a high school diploma, enrollment in and graduation from a 4‐year college, smoker or nonsmoker, alcohol intake, and (social networks) if immediate family members, relatives, or friends have sensitive diseases—could be fully extracted and directly used from EMRs. However, for features such as the number of health workers in the community, availability of telehealth services or mobile health units, and without convenient transportation, only minimal data are available. Detailed information can be found in Table 2.

**Table 2:** SDoH information completeness and availability levels in Chinese EMRs.

Feature	Max completeness level	Max availability level
Pay	None	None
Without health insurance	3	3
Paid sick leave	None	None
Paid parental leave	None	None
Retirement savings	None	None
Without household food insecurity and hunger	1	2
Without trouble paying rent	None	None
Moving frequency	None	None
Spend the bulk of household income on housing or not	None	None
Defined as poverty or not	1	3
Without access to early education programs	3	3
Without a high school diploma	3	3
Enrollment in and graduation from a 4‐year college	3	3
Oral literacy (listening and speaking skills)	None	None
Print literacy (writing and reading skills)	None	None
Numeracy (the ability to understand and work with numbers)	None	None
Cultural and conceptual knowledge	None	None
Number of health workers in community	1	1
Availability of telehealth services or mobile health unit	1	1
Without convenient transportation	1	1
Number of grocery stores, food services, or farmer markets	1	1
Distance from the nearest grocery stores, food services, or farmer markets	1	1
Variety of grocery stores, food services, or farmer markets	1	1
Quality of grocery stores, food services, or farmer markets	1	1
Costs of daily foods or meals	None	None
Access to reliable transportation	1	1
Participate in community food support programs or not	None	None
Water quality	1	1
Air quality	1	1
Noise levels	1	1
Proximity to hazardous waste sites	1	1
Severe weather and climate change	1	1
Physical conditions of a person's home	1	1
Surrounding neighborhood	1	1
Experience bullying or cyberbullying, abuse, or witnessing violence in a variety of settings or not	None	None
Exposed to intimate partner violence or not	None	None
Exposed to elder abuse or not	None	None
Exposure to crime or violence in one's community	None	None
In the less privileged groups or residential segregation or not	None	None
Discrimination based on age, gender, sexuality, gender identity, and disabilities	None	None
Number of community groups and community activities	None	None
Existence/frequency of other formal or informal activities	None	None
The amount of social capital a community has	None	None
(Social networks) if immediate family members, relatives, or friends have sensitive diseases	3	3
Sources of social support	None	None
Without a history of incarceration	None	None
There is an incarcerated person within household or not	None	None
Gender, age, ethnicity, marital status, number of family members, etc.	1	3
Smoker or nonsmoker	3	3
Alcohol intake	3	3

Abbreviations: EMR, electronic medical record; SDoH, social determinants of health.

### 3.4 Extraction and Analysis of SDoH Information in EMRs

Regarding the five features of SDoH information (namely, (1) without health insurance, (2) education level, (3) if immediate family members, relatives, or friends have sensitive diseases, (4) smoker or nonsmoker, and (5) alcohol intake), all of these could be directly obtained from the current EMRs with the highest corresponding information quality. We extracted these five types of features across the entire database to construct an SDoH features table, and we also included some basic patient information such as information from hospitals and diseases.

**Figure 2:**
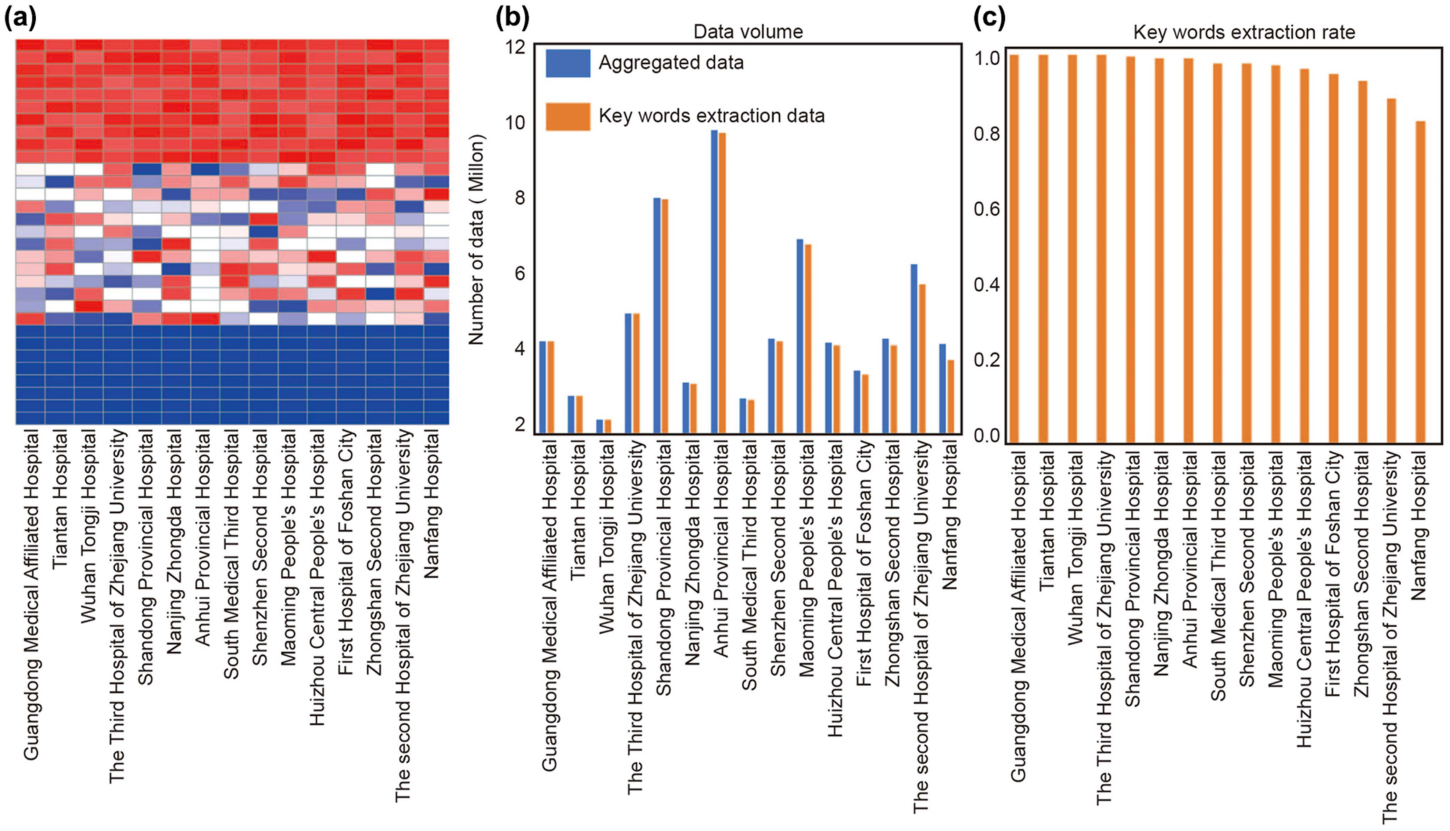
Comparison of SDoH information in EMRs of different hospitals. (a) The heat map depicts the proportion of diverse SDoH factors existing in EMRs across various hospitals. A shade closer to red indicates a frequency nearer to 1 and a shade closer to blue indicates a frequency nearer to 0. (b) Bar plot showing the total number of EMRs in different hospitals (blue) and number of cases with SDoH information after keyword extraction (orange). (c) Bar chart showing the proportion of SDoH‐containing patient data at different hospitals. EMR, electronic medical record; SDoH, social determinants of health.

By analyzing the source centers of these data, we found that not all EMRs provide comprehensive data for SDoH. We statistically analyzed the frequency of occurrence of five types of SDoH information within patient EMRs in 15 hospitals (> 50,000 patients) (Figure 2a). This finding indicates that a severe imbalance exists in the collection of SDoH information in Chinese EMRs at present. It is suggested that hospitals gather these data through questionnaires or other complementary approaches in the future. However, we also observed that the proportion of extractable SDoH information in the EMRs of different hospitals varied, and valid SDoH information could not be extracted from some EMRs (Figure 2b). Through analysis of these medical records, we discovered that this phenomenon is associated with the level of detail in hospital doctors' records and the writing standards of medical records. Specifically, the data extraction ratio of Guangdong Medical Affiliated Hospital was the highest, reaching 99.9%, whereas that of Southern Hospital was 82.8% (Figure 2c). This finding emphasizes the importance of developing uniform EMR writing norms across hospitals.

### 3.5 Lessons Learned

In the initial phase of our research, we attempted to directly extract desired SDoH factors from EMR data using NLP techniques such as entity recognition and relationship extraction. However, we observed that these features occurred with notably low frequency in Chinese EMRs, as the primary purpose of China's EMR systems remains clinical diagnosis/treatment and administrative management rather than public health objectives. Traditional NLP methods proved less effective in processing such information. Consequently, we ultimately shifted our focus to evaluating both the feasibility of extracting these features from EMRs and the practical utility of extracted information for public health recommendations. In subsequent research phases, we plan to explore advanced technical approaches including large language models (LLMs) to address these challenges, discussed further below.

## 4 Discussions

### 4.1 Principal Findings

Approximately half of SDoH features can be extracted from Chinese EMRs, but most of these features only reflect a small portion of the SDoH information. However, owing to the structured nature of Chinese EMRs and specialized NLP processing techniques, many pieces of information can be used directly, without the need for additional complex processing. Given the medical nature of EMRs, high‐quality extraction of SDoH features is primarily concentrated in the categories of Education access and quality and Other individual‐level SDoH. Other types of SDoH features can only be extracted to a minimal extent. Nevertheless, because EMRs contain a wealth of patient health outcome data, they offer an excellent resource for conducting research on the correlations between SDoH and health outcomes.

We specifically explored the feasibility of extracting SDoH features from Chinese EMRs. Coupled with the extensive and highly efficient data exchange network among Chinese government departments, we managed to extract 25 features of six types of SDoH information from these fields. However, because Chinese medical institutions do not specifically gather SDoH data, we could only extract 10 pieces of SDoH information from most patients, which is far fewer than the 50 SDoH characteristics we have summarized. However, even for patients from whom SDoH information such as educational level and neighborhood environment could be extracted, there is currently no uniform indicator to evaluate this information. Ambiguity of the standards results in reduced validity of the information. Therefore, we propose that hospitals and other institutions effectively collect the aforementioned information in a reasonable manner and formulate corresponding SDoH information grading standards based on its distribution characteristics. Additionally, certain information, such as numeracy, reading and writing skills, and community‐related data, cannot be directly extracted from EMRs but must rely on indirect inferences from SDoH‐related fields. This approach may carry a high risk of inaccurate information. It is expected that in the future, hospitals and other relevant institutions in China will adopt appropriate methods to directly access these data, which will greatly facilitate more comprehensive treatment and care for patients from an environmental and social perspective. We believe that this process will rapidly advance the development of medical economics in China.

In the era of digital medical information, integrating NLP technology to establish indicators and information on critical social factors is essential. We evaluated the feasibility and effectiveness of extracting a range of SDoH features (such as income, education, housing, food insecurity, environmental conditions, and social support) from Chinese EMRs. Fifty percent of SDoH features can be extracted from EMR data, but the completeness of the information is limited, as it does not fully reflect all SDoH features. However, by relying on NLP technology and the data model structure of EMRs, most information can be used directly as structured data without the need for additional processing. The quality of the extracted information varies widely. In regions and hospitals with standardized documentation and high‐quality data, over 18 features could be extracted for nearly all patients. In other regions, only up to 10 features could be extracted. Conducting SDoH feature research based on EMRs is beneficial as it permits direct exploration of the impact these features have on human health.

### 4.2 Comparison to Prior Work

The primary sources of SDoH information, a considerable portion of which mirrors personal data and the degree of social healthcare services, can be found in the EMRs using RWD. With continued growth and development of the economy and civil society, SDoH factors affecting health have attracted increasing attention. The annual County Health Rankings, which gauge the impact of a wide range of health factors, indicate that social and economic factors contribute to 40% of health outcomes. However, current studies on SDoH often exhibit several deficiencies: First, studies tend to analyze the influence of socioeconomic factors on the development of specific diseases, without research and analysis based on a national database. Regarding index extraction, manual or semi‐automatic approaches are adopted to determine the indices, and there is no national standard index system for diseases. Finally, for model training, there are no case analyses founded upon big data.

In the present study, we established a contextually adapted SDoH framework for China's healthcare system and conducted the first systematic feasibility investigation of SDoH extraction from EMRs in Chinese clinical settings. This landmark study demonstrates the viability of leveraging national‐scale big data samples to analyze critical socioeconomic health determinants, generating empirically grounded evidence for public health governance frameworks while simultaneously advancing semantic mining capabilities in clinical narratives. We anticipate that this methodological innovation will catalyze a paradigm shift in health economic analytics through data‐driven healthcare policy formulation.

### 4.3 Limitations

This study had several limitations. First, from the perspective of SDoH indices, the establishment of a national SDoH index is still pending, and there has been no comprehensive or pilot study on the impact of social and economic factors. This area is in its nascent stage and requires further promotion and development. Although our preliminary exploration of an SDoH index may have methodological limitations in scope, we urge Chinese researchers to intensify multidisciplinary investigations and scholarly discourse regarding these critical health determinants. Such collective intellectual engagement will prove instrumental in systematically refining this essential dimension of China's healthcare informatics infrastructure.

Second, from the perspective of extractability, half of the SDoH features can be directly extracted from EMRs, but the strength of the extracted information is limited. This presents a substantial challenge regarding standards and consistency in completing Chinese EMRs. It must be acknowledged that the clinical‐administrative orientation inherent in Chinese EMRs constitutes the primary etiological factor underlying this phenomenon. Implementing paradigm‐shifting initiatives through public health policy channels to optimize EMR architecture for socioeconomic health surveillance could engender transformative synergies between clinical documentation practices and population health analytics.

Finally, differences in data standards lead to variations in the extraction of information across different regions and healthcare institutions. The inherent issue of data standard discrepancies in China's healthcare systems makes developing EMR standards that align with China's data characteristics while incorporating international standards—and continuously promoting them across regional healthcare institutions—a crucial task that constitutes the primary focus of our research team.

### 4.4 Future Directions

The primary purpose of this study was to develop a Chinese version of the SDoH features table and explore the feasibility of extracting SDoH information from China's vast EMR resource. Through our attempts, we confirmed that EMRs contain a large amount of SDoH information, which can also be extracted through corresponding technical means. Our subsequent research is mainly focused on the following two directions.

We aim to expand and optimize the SDoH features table. The formulation of this table mostly relies on our literature review and partial data research, lacking judgment of its effectiveness in practical applications. We will apply this table in different institutions and regions and determine its effectiveness, while making targeted improvements.

We also aim to conduct comprehensive extraction of SDoH information in EMRs using technical means. LLM technology has developed rapidly, bringing improvements in model capabilities as well as the optimization of various model application methods and techniques such as Prompt‐Retrieval‐Augmented Generation. We will also try to use LLMs for the comprehensive extraction of SDoH information. This will be a relatively new direction that could help to address current technical difficulties.

## 5 Conclusions

According to the situation in China and relevant literature reviews, we proposed a classification of SDoH features specific to China and explored the feasibility of extracting these features from Chinese EMRs. We demonstrated that extracting SDoH features from Chinese EMRs is feasible, and we were able to extract SDoH information from large existing databases to support future research. We aim to lay a strong foundation for the future study of SDoH in China, facilitating more comprehensive and in‐depth research in this field. The results of our study can inform government and relevant departments regarding the importance of SDoH interventions. Our study findings can provide scientific evidence and policy support to improve social health outcomes. Our research offers a theoretical framework for effectively identifying and addressing the social determinants affecting human health, contributing to the refinement of public health policies and health management strategies as well as encouraging society to pay greater attention to the issue of health inequities.

## Author Contributions

**Mengchun Gong:** methodology, conceptualization, project administration, funding acquisition, formal analysis, writing – review and editing, resources. **Zihao Ouyang:** methodology, software, data curation, visualization, conceptualization, writing – original draft, writing – review and editing, validation. **Dandan Ma:** writing – review and editing, conceptualization, methodology, formal analysis. **Qilin Wang:** investigation, data curation. **Endi Cai:** investigation, data curation. **Chao Liu:** investigation, data curation, supervision. **Yue Yu:** data curation, investigation. **Jingdong Yan:** methodology, supervision, resources, project administration. **Sheng Nie:** methodology, supervision, project administration, funding acquisition. **Lin Lin:** conceptualization, supervision, writing – review and editing.

## Ethics Statement

The study protocol was reviewed and approved by the Medical Ethics Committee of Nanfang Hospital, Southern Medical University (approval no. NFEC‐2019‐213). The study was conducted in accordance with the Declaration of Helsinki (as revised in 2013) and the applicable institutional and national ethical guidelines.

## Consent

The requirement for individual informed consent was waived by the Medical Ethics Committee of Nanfang Hospital, Southern Medical University due to the retrospective nature of the study and the use of de‐identified electronic medical record data.

## Conflicts of Interest

Mengchun Gong serves as the Vice President of DHC Technologies Ltd. Chao Liu serves as Head of the Medical Research Department at DHC Technologies Ltd. Zihao Ouyang, Qilin Wang, Endi Cai, and Yue Yu are medical researchers in the Medical Research Department at DHC Technologies Ltd. The other authors declare no conflicts of interest.

## Supporting information


Supporting File 1

Supporting File 2

## Data Availability

The data that support the findings of this study are available on request from the corresponding author. Owing to the sensitivity of the hospital data, the dataset cannot be made publicly available.
