# Supplementary material for: Feasibility Assessment of Extracting Social Determinants of Health From Electronic Medical Records: A Multicenter Study Across Chinese Healthcare Institutions
Source: Health Care Sci. 2026 Aug 31:10.1002/hcs2.70094. Online ahead of print. doi: 10.1002/hcs2.70094 (PMC13528596; doi:10.1002/hcs2.70094)
Supplement: Supplementary file 1 [file HCS2-9999-0-s002.docx]

**Supplementary Table S1**. CDM of CRDS.

| **Chinese Renal Disease System (CRDS) data model** | | |
| --- | --- | --- |
| **Type** | **Table name** | **Description** |
| **Patients visit information** | IM_PERSON | Patients basic information table |
|  | IM_VISIT | Patients visit information table |
| **Patients clinical information** | IM_CONDITION | Diagnosis information table |
|  | IM_DRUG | Drug information table |
|  | IM_MEASUREMENT | Measurement information table |
|  | IM_ PROCEDURE_OCCURRENCE | Operation information table |
|  | IM_MEDICAL_TECHNOLOGY | Medical imaging information table |
|  | IM_DEATH | Death information table |
|  | IM_NOTE | Record text information table |
|  | IM_SIGNS | Signs information table |
| **NLP information** | chiefComplaint | Chief complaint information table |
|  | drinkSmokeHistory | Drink and smoke history information table |
|  | toxicHistory | Toxic information table |
|  | diseaseName | Disease information table |
|  | diseaseOperation | Surgical procedure information table |
|  | allergy | Allergy history information table |
|  | familyHistory | Family history information table |
|  | marriageHistory | Marriage and childbirth history information table |
|  | vital_Signs | Vital signs information table |

Abbreviations: CRDS, Chinese Renal Disease System; NLP, natural language processing.

**Supplementary Table S2**. SDoH features.

| **SDoH type** | **Subtype** | **Feature** |
| --- | --- | --- |
| 1. Economic stability | Income | Pay |
|  | Job benefits | Without health insurance |
|  |  | Paid sick leave |
|  |  | Paid parental leave |
|  |  | Retirement savings |
|  | Nutrition and healthy eating | Without household food insecurity and hunger |
|  | Housing instability | Without trouble paying rent |
|  |  | Moving frequency |
|  |  | Number of properties owned by the family |
|  | Poverty | Defined as poverty or not |
| 2. Education access and quality | Early childhood development and education | Without access to early education programmes |
|  | High school graduation | Without a high school diploma |
|  | Enrolment in higher education | Enrolment in and graduation from a 4-year college |
|  | Language and literacy | Oral literacy (listening and speaking skills) |
|  |  | Print literacy (writing and reading skills) |
|  |  | Numeracy (the ability to understand and work with numbers) |
|  |  | Cultural and conceptual knowledge |
| 3. Health care access and quality | Access to primary care | Number of health workers in community |
|  | Access to health services | Availability of telehealth services or mobile health unit |
|  |  | Without convenient transportation |
| 4. Neighbourhood and built environment | Access to foods that support health dietary pattern | Number of grocery stores, food services or farmer markets |
|  |  | Distance from the nearest grocery stores,  Food services or farmer markets |
|  |  | Variety of grocery stores, food services or farmer markets |
|  |  | Quality of grocery stores, food services or farmer markets |
|  |  | Costs of daily foods or meals |
|  |  | Access to reliable transportation |
|  |  | Participate in community food support programmes or not |
|  | Environmental conditions | Water quality |
|  |  | Air quality |
|  |  | Noise levels |
|  |  | Proximity to hazardous waste sites |
|  |  | Severe weather and climate change |
|  | Quality of housing | Physical conditions of a person’s home |
|  |  | Surrounding neighbourhood |
|  | Crime and violence | Experience bullying or cyberbullying, abuse, or witnessing violence in a variety of settings or not |
|  |  | Exposed to intimate partner violence or not |
|  |  | Exposed to elder abuse or not |
|  |  | Exposure to crime or violence in one’s community |
| 5. Social and community context | Discrimination | In the less privileged groups or residential segregation or not |
|  |  | Discrimination based on age, gender, sexuality, gender identity and disabilities |
|  | Civic participation | Number of community groups and community activities |
|  |  | Existence/frequency of other formal or informal activities |
|  | Social cohesion | The amount of social capital a community has |
|  |  | (Social networks) if immediate family members, relatives or friends has sensitive diseases |
|  |  | Sources of social support |
|  | Incarceration | Without a history of incarceration |
|  |  | There is an incarcerated person within household or not |
| 6. Other individual-level SDoH | Socio-demographical characteristics | Gender, age, ethnicity, marital status, number of family members, etc. |
|  | Behavioural indicators | Smoker or non-smoker |
|  |  | Alcohol intake |

Abbreviation: SDoH, social determinants of health.

**Supplementary Table S3**. SDoH information contained level in China EMR.

| Feature | Max completeness level | Max availability level |
| --- | --- | --- |
| Pay | None | None |
| Without health insurance | 3 | 3 |
| Paid sick leave | None | None |
| Paid parental leave | None | None |
| Retirement savings | None | None |
| Without household food insecurity and hunger | 1 | 2 |
| Without trouble paying rent | None | None |
| Moving frequency | None | None |
| Spend the bulk of household income on housing or not | None | None |
| Defined as poverty or not | 1 | 3 |
| Without access to early education programmes | 3 | 3 |
| Without a high school diploma | 3 | 3 |
| Enrolment in and graduation from a 4-year college | 3 | 3 |
| Oral literacy (listening and speaking skills) | None | None |
| Print literacy (writing and reading skills) | None | None |
| Numeracy (the ability to understand and work with numbers) | None | None |
| Cultural and conceptual knowledge | None | None |
| Number of health workers in community | 1 | 1 |
| Availability of telehealth services or mobile health unit | 1 | 1 |
| Without convenient transportation | 1 | 1 |
| Number of grocery stores, food services or farmer markets | 1 | 1 |
| Distance from the nearest grocery stores, food services or farmer markets | 1 | 1 |
| Variety of grocery stores, food services or farmer markets | 1 | 1 |
| Quality of grocery stores, food services or farmer markets | 1 | 1 |
| Costs of daily foods or meals | None | None |
| Access to reliable transportation | 1 | 1 |
| Participate in community food support programmes or not | None | None |
| Water quality | 1 | 1 |
| Air quality | 1 | 1 |
| Noise levels | 1 | 1 |
| Proximity to hazardous waste sites | 1 | 1 |
| Severe weather and climate change | 1 | 1 |
| Physical conditions of a person’s home | 1 | 1 |
| Surrounding neighbourhood | 1 | 1 |
| Experience bullying or cyberbullying, abuse, or witnessing violence in a variety of settings or not | None | None |
| Exposed to intimate partner violence or not | None | None |
| Exposed to elder abuse or not | None | None |
| Exposure to crime or violence in one’s community | None | None |
| In the less privileged groups or residential segregation or not | None | None |
| Discrimination based on age, gender, sexuality, gender identity and disabilities | None | None |
| Number of community groups and community activities | None | None |
| Existence/frequency of other formal or informal activities | None | None |
| The amount of social capital a community has | None | None |
| (Social networks) if immediate family members, relatives or friends has sensitive diseases | 3 | 3 |
| Sources of social support | None | None |
| Without a history of incarceration | None | None |
| There is an incarcerated person within household or not | None | None |
| Gender, age, ethnicity, marital status, number of family members, etc. | 1 | 3 |
| Smoker or non-smoker | 3 | 3 |
| Alcohol intake | 3 | 3 |

Abbreviations: EMR, electronic medical record; SDoH, social determinants of health.
